# Solitary restriction endonucleases in prokaryotic genomes

**DOI:** 10.1093/nar/gks853

**Published:** 2012-09-08

**Authors:** Anna S. Ershova, Anna S. Karyagina, Mikhail O. Vasiliev, Alexander M. Lyashchuk, Vladimir G. Lunin, Sergey A. Spirin, Andrei V. Alexeevski

**Affiliations:** ^1^Department of Mathematical Methods in Biology, Belozersky Institute of Physico-Chemical Biology, Lomonosov Moscow State University, 119992, ^2^Laboratory of Biologically Active Nanostructures, Gamaleya Institute of Epidemiology and Microbiology, Russian Federation Ministry of Health and Social Development, 123098, ^3^Laboratory of Genome Analysis, Institute of Agricultural Biotechnology, Russian Academy of Agricultural Sciences, 127550, ^4^Department for Computational Mathematics, Moscow Institute of Physics and Technology, Dolgoprudny, 141700, ^5^Laboratory of Molecular Diagnostics and Genetic Engineering, Institute of Agricultural Biotechnology, Russian Academy of Agricultural Sciences, 127550, ^6^Sector of Applied Informatics, Scientific Research Institute for System Studies, Russian Academy of Sciences, 117218 and ^7^Faculty of Bioengineering and Bioinformatics of Lomonosov Moscow State University, 119991, Moscow, Russia

## Abstract

Prokaryotic restriction-modification (R-M) systems defend the host cell from the invasion of a foreign DNA. They comprise two enzymatic activities: specific DNA cleavage activity and DNA methylation activity preventing cleavage. Typically, these activities are provided by two separate enzymes: a DNA methyltransferase (MTase) and a restriction endonuclease (RE). In the absence of a corresponding MTase, an RE of Type II R-M system is highly toxic for the cell. Genes of the R-M system are linked in the genome in the vast majority of annotated cases. There are only a few reported cases in which the genes of MTase and RE from one R-M system are not linked. Nevertheless, a few hundreds solitary RE genes are present in the Restriction Enzyme Database (http://rebase.neb.com) annotations. Using the comparative genomic approach, we analysed 272 solitary RE genes. For 57 solitary RE genes we predicted corresponding MTase genes located distantly in a genome. Of the 272 solitary RE genes, 99 are likely to be fragments of RE genes. Various explanations for the existence of the remaining 116 solitary RE genes are also discussed.

## INTRODUCTION

Restriction endonucleases (REs) are components of prokaryotic restriction-modification (R-M) systems. Except in certain types of R-M systems, REs recognize non-methylated DNA sites with a certain sequence and hydrolyze DNA within the sites or in their vicinity. DNA methyltransferase (MTase), other mandatory component of R-M system, methylates DNA bases within the recognition sites of the same sequence, and thus prevent DNA hydrolysis by REs.

R-M systems protect bacteria or archeae from the penetration of a foreign DNA, particularly the DNA of bacteriophages, because foreign DNA, as a rule, is not methylated in specific sites. R-M systems are also thought to be the moderators of evolution due to accidental cleavage of self DNA by REs and the consequent ligation of fragments not necessarily in initial order (1,2).

Despite the absence of their own mechanisms of mobility, R-M systems are considered selfish genetic elements (1). R-M systems are often found on mobile genetic elements (plasmids, transposable elements, prophages, genomic islands) (1).

R-M systems are classified into four types (3,4), which differ in the structure of active enzyme complexes, in the distance between a recognition site and a site of hydrolysis and in the requirement for cofactors. R-M systems comprising REs that recognize non-modified sites are of Types I, II (excluding subtype IIM), and III (4). In typical Type I R-M systems, the DNA is hydrolyzed by a complex of two MTases, two REs and an S-subunit, the last being a DNA-recognizing protein. MTases and REs of Type II R-M systems, as a rule, operate as separate proteins. R-M systems of subtype IIG consist of one protein with fused MTase and RE domains. Type III systems function as a complex of two MTases and two REs, and only MTases have DNA recognition domains (4).

REs of subtype IIM (5) and Type IV (3) cleave-methylated DNA. No other genes are required for the majority of these R-M systems.

With some exceptions, the genes of all characterized R-M systems are linked on a chromosome or a plasmid (1,6,7). Co-localization of the genes, often observed for functionally connected genes, is in agreement with the selfish behavior of R-M systems (1). At the same time, there are no visible obstacles to R-M systems functioning if their genes are distantly located in a genome (1). The only known examples of R-M systems with separated RE and MTase genes are functional Type I R-M systems from three strains of *Staphylococcus aureus*, in which linked MTase and S-subunit genes are located far from the corresponding RE genes (8,9). Type I R-M systems with separated genes of the S-subunit and linked RE and MTase genes have been found in *Lactococcus lactis* and *Mycoplasma pneumonia* (10,11). The products of the linked RE and MTase chromosomal genes form functional complexes with S-subunits encoded by plasmids, as well as complexes with another S-subunit, whose gene is co-localized with the RE and MTase genes.

In the Restriction Enzyme Database (REBASE; release of February 2010) we found 276 RE genes of Types I–III that have no corresponding MTase genes or have only corrupted corresponding MTase genes (with frameshift or truncated ones). The products of RE genes retaining DNA recognition and cleavage activity, and lacking a corresponding MTase, might be highly toxic for the cell (12).

We suppose that if these RE genes are produced into functional endonucleases, then the host genome should contain MTase genes with recognition sequences covering the solitary RE’s recognition sequence, thus protecting the host cell. We used the comparative genomics approach to find such MTases. For some solitary REs we suggested paired MTases. However, we failed to explain all cases of solitary REs by our method. In the Discussion section we consider also other possible reasons of the existence of solitary REs based on previously published analogous cases.

## MATERIALS AND METHODS

### R-M systems and genomes

Data on Types I, II and III R-M systems in 1040 complete bacterial and archaeal genomes were downloaded from REBASE (14,15), released February 2010. Prokaryotic genome sequences and their annotations were downloaded from the National Center for Biotechnology Information (NCBI; ftp://ftp.ncbi.nih.gov/genomes/).

Annotations of R-M genes as well as recognition sequences of proteins were taken from REBASE. The accepted REBASE methods of R-M gene and recognition sequence predictions by similarity are described in (14). R-M genes, annotated as ‘pseudo’ in the annotations of NCBI genome entries, were marked as corrupted.

R-M systems, including both non-corrupted RE and MTase genes, were marked as ‘complete’ R-M systems. All remaining R-M systems were marked as ‘incomplete’.

Supplementary Table S1 lists all analysed genomes.

### Solitary RE

All incomplete R-M systems with non-corrupted RE genes were selected. For each of them, all MTase-like open reading frames (ORFs) in the vicinity of the RE gene (4 kb upstream and downstream of the translation start and stop codons of the RE gene) were found using TBLASTN (16), with *E* < 0.01, and the program *getorf* from the EMBOSS package. All MTases domains were given on input. No new ORFs, except for those annotated in REBASE, were identified.

Thus, we denote the RE as solitary if its gene has no corresponding non-corrupted MTase genes in the vicinity.

Besides the RE genes already excluded as corrupted (according to genome entry annotations), we also marked genes that are presumably fragments of functional RE genes. The latter were determined by comparing the solitary RE protein sequence with sequences of all its homologs among REs from complete R-M systems with amino acid (aa) identity >20% in BLASTp alignment. If the solitary RE was shorter by 20% or more than any homolog, then the solitary RE was marked as ‘probably truncated RE’. Supplementary Table S2 presents all analysed solitary REs.

### Comparison of solitary REs with methyl-directed REs (Type IIM and Type IV)

We checked that selected solitary REs were not likely to be methyl-directed REs, using sequence analysis in the following manner. A BLASTp search (*E* < 0.001) of all Type IIM and Type IV RE protein sequences against all RE sequences downloaded from REBASE was performed. No solitary RE hits of Type IIM sequences were found.

All hits of Type IV REs, including hits of solitary REs, were clustered by >40% of aa identity over 60% of the length of each sequence. Only two clusters contained both solitary REs and Type IV REs. The sequences of these two clusters were aligned, and phylogenetic trees were constructed by neighbor joining algorithm. A bootstrap analysis was performed. Supplementary Figures S1 and S2 present the results.

### Orthologous REs and MTases

We used the best bidirectional hits method (17) to search for orthologs. This method, as originally designed, does not normally take similarity level into account, so we had to add this functionality to select the closest orthologs. Two REs from different genomes were considered orthologous if their aa sequences had >40% of aa identity over 80% of the length of the longer sequence. For MTases, the threshold of >50% of aa identity over 80% of length was used due to the typically higher sequence similarity of MTases. According to these criteria, a studied protein usually has no more than one ortholog in a genome.

### Orthologous R-M systems

Two pairs of REs and MTases encoded in two different genomes are called orthologous if their REs and MTases are orthologs. An RE/MTase pair that was not previously annotated in REBASE as an R-M system, but has orthologous RE/MTase pairs in other genomes, is considered a putative new R-M system.

**Figure 1.**
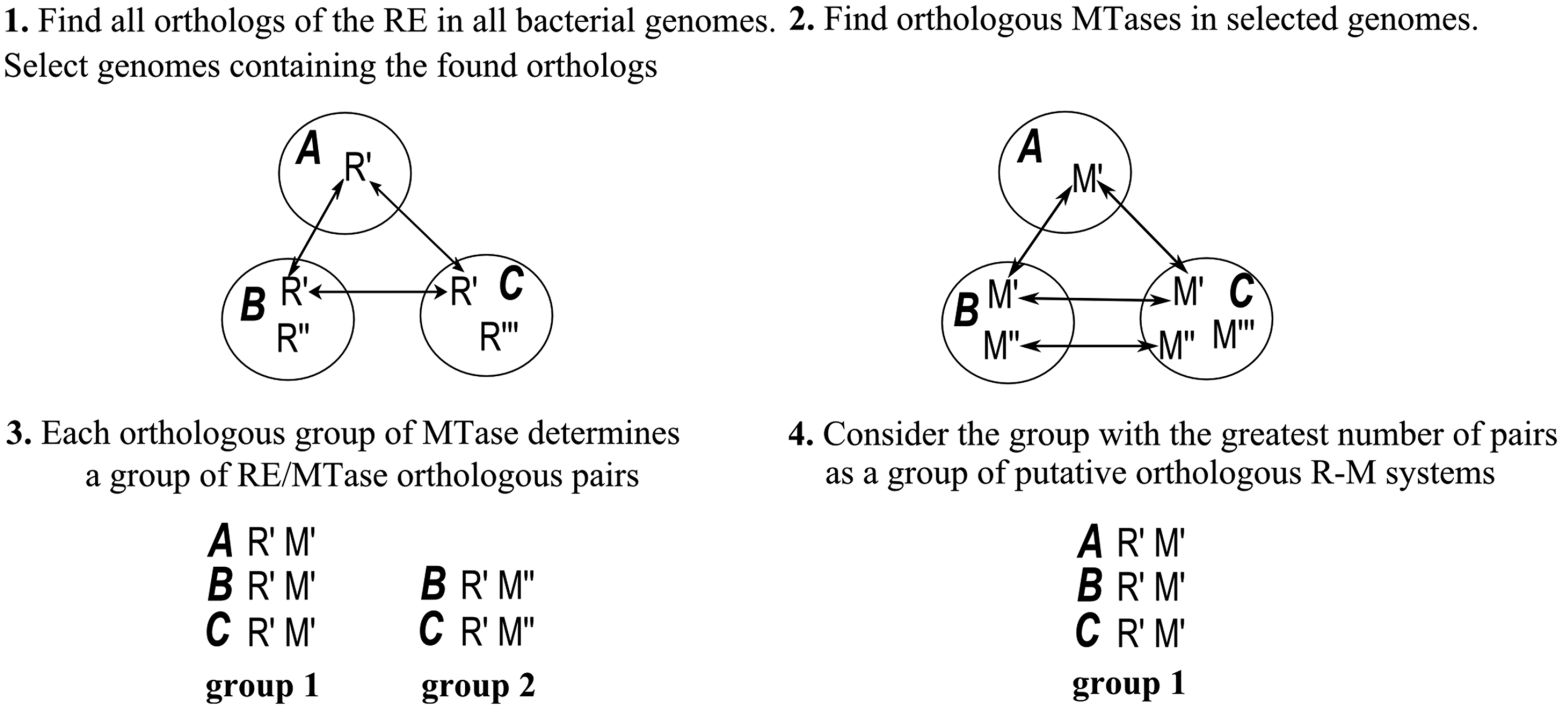
The algorithm of the orthologous R-M systems search. Big circles denote genome, small circles denote MTase, squares denote RE and arrows connect orthologs.

Figure 1 describes the algorithm of the orthologous R-M system identification.

### Separated R-M systems

The simplest way to predict the MTase corresponding to a given solitary RE is to compare a recognition site of the solitary RE and the recognition sites of all MTases encoded by the same genome. Unfortunately, for the majority of REs and MTases, the recognition DNA sequences are unknown, or are predicted by sequence similarity to enzymes with known recognition specificity. That is why we predicted a corresponding MTase by the above-described comparative genomics approach (see Orthologous R-M system section).

In the case of a distant location of such RE and MTase genes in a genome, we assume them to be a separated R-M system. In a number of cases, this prediction is supported by additional arguments (see ‘Results’ section).

We denote a putative R-M system as ‘separated’ if it is a member of a group of orthologous R-M systems and the distance between the genes coding for RE and MTase is >4 kb. Supplementary Table S5 presents the complete list of detected groups of all orthologous R-M systems that include separated R-M systems. Supplementary Alignments present the sequence alignments for corresponding proteins.

### Genomic neighborhood analysis of the RE and MTase genes of separated R-M systems

We analysed ORFs in the 20 kb vicinity of RE or MTase genes to reveal mobile genetic elements (18): insertion sequence elements, the genes of transposases, integrases and phage-related proteins.

The corresponding genes were found by the genome annotations. If we found a mobile genetic element, we searched for similar elements in other genomes of the corresponding orthologous group of R-M systems (BLAST, *E* < 0.01).

## RESULTS

### Identification of solitary RE genes in complete genomes of bacteria and archea and their classification by probably

We identified 272 solitary REs genes in the genomes from list of 1040 genomes. We have several explanations for their existence in genome. Taking into account that only Type II REs are functional without corresponding MTase, we describe safe and useless solitary REs (of Type I and III) and possibly toxic (of Type II) REs separately.

As one can expect, we have found much more safe REs than probably toxic ones (Table 1).

**Table 1.** Summary of full-length solitary REs from prokaryotic genomes

Type of solitary RE	Probably truncated REs	REs that are parts of putative-separated R-M systems	REs with nearby location of corrupted MTase genes	REs for which no predicted paired MTases were found	REs without orthologs in other genomes	Total
I	79	38 (3)^a^	29 (17)	10 (7)	3 (3)	159
II	16	19 (6)	11 (10)	21 (10)	7 (7)	74
III	4	0	34 (13)	1 (1)	0	39
Total	99	57 (9)	74 (39)	32 (18)	10 (10)	272

^a^The numbers in parentheses indicate the number of groups of orthologous REs.

The initial list of 272 solitary RE genes was reduced to 173 genes by excluding genes likely to be fragments of full-length genes (probably truncated REs in Table 1) (see Materials and Methods section). For these REs, a search of orthologs with sufficient similarity of sequences of the REBASE list of REs was performed. We excluded from the analysis 10 REs for which no orthologs with sufficiently similar sequences were found, thus making it impossible to find a corresponding MTase by our method (for the list of all possible cognate MTases from the genome for these REs, see Supplementary Table S6).

The remaining 163 solitary REs fall into 58 orthologous groups that include two or more REs. Of these groups, 26 include two or more solitary REs (see Supplementary Table S2). We performed search of corresponding MTase for these 163 solitary REs. Thus, all solitary REs we divided into three classes (Table 1): (i) REs that are parts of putative-separated R-M systems; (ii) REs with nearby location (4 kb upstream and downstream of the translation start and stop codons of the RE gene) of corrupted MTase genes with translation frameshift or unexpected stop codon; and (iii) REs for which no paired MTases or corrupted MTase genes in the vicinity were found in their genomes.

### Comparison of the identified solitary REs with methyl-directed REs

To try to decrease the possibility that the solitary REs found are actually methyl-directed REs and, thus, there is no need for them to have corresponding MTases, we compared the sequences of solitary REs with the sequences of known methyl-directed REs. A BLAST search showed no hits of solitary REs among methyl-directed REs of Type IIM RE sequences.

Two clusters of homologs of methyl-directed Type IV REs contained solitary REs (see Materials and Methods section and Supplementary Figures S1 and S2). Genomic context of all REs from cluster 1 includes additional linked RE. Such pairs of two-linked REs were studied in (19,20). It was experimentally proven for one of them, LlaJI from *L. lactis,* that active Type II RE is formed as heterodimer of products of two RE genes (24). In keeping with works (24,25) we suppose that all REs from cluster 1, including those annotated as Type IV REs, are actually components of Type II REs, see Supplementary Figure S1. Sequence alignment and phylogenetic tree analysis, accomplished with bootstrap analysis, revealed that solitary Type II REs from cluster 2 should not be Type IV ones, see Supplementary Figure S2.

Thus we cannot say that any one of our solitary REs is similar to known methyl-directed one, but we cannot exclude that some of them are actually methyl-directed.

### Groups of orthologous R-M systems

For the 163 solitary REs a search of cognate MTases was performed (see Materials and Methods section). We found putative corresponding MTases for 57 solitary REs. Thus, these 57 REs are members of putative-separated R-M systems. These R-M systems belong to 11 groups of orthologous R-M systems of Types I and II (Table 2). Separated R-M systems of Type III were not identified.

**Table 2.** The list of orthologous R-M systems groups, which include putative separated R-M systems

Number of group	Type	Taxon	Number of R-M systems	Number of R-M systems annotated in REBASE	Number of separated R-M systems	Number of solitary REs
1	II	*Bordetella*: three species	3	2	1	1
2	II	Proteobacteria: three classes	5	2	3	3
3	II	α-Proteobacteria: four orders	5	0	5	5
4	II	*Clostridium perfringens*: three strains	3	0	3	3
5	II	*Fibrobacter succinogenes*: two strains	2	0	2	2
6	II	*Bacteroides*: two species	2	0	2	2
7	II	*Bacteroides*: three species	3	0	3	3
8	I	Archaea, Bacteria	79	29	50 ^a^	27 ^b^
9	I	Archaea, Bacteria	101	100	4	4
10	I	Archaea, Bacteria	24	23	1	1
11	I	Archaea, Bacteria	128	122	6	6

^a^The functionality of separated R-M systems from three strains of *S. aureus* has been proved experimentally [9]; see also text.

^b^Of the 27 solitary REs, 25 are from strains of *S. aureus*, 23 *S. aureus* strains contain 1 RE gene and 2 cassettes of genes of MTase and S-proteins. This is why the number of separated R-M systems exceeds the number of solitary REs.

With two exceptions, groups of orthologous R-M systems that include predicted separated systems consist of R-M systems of organisms from essentially distant taxons: different species, classes, orders or even kingdoms. Supplementary Table S5 presents the complete list of all orthologous R-M systems that include separated R-M systems.

All separated R-M systems are predicted on the basis of aa sequence similarity and have different degrees of reliability. We consider that R-M systems for which orthologous ordinary (not separated) R-M systems are found as more reliable.

**Figure 2.**
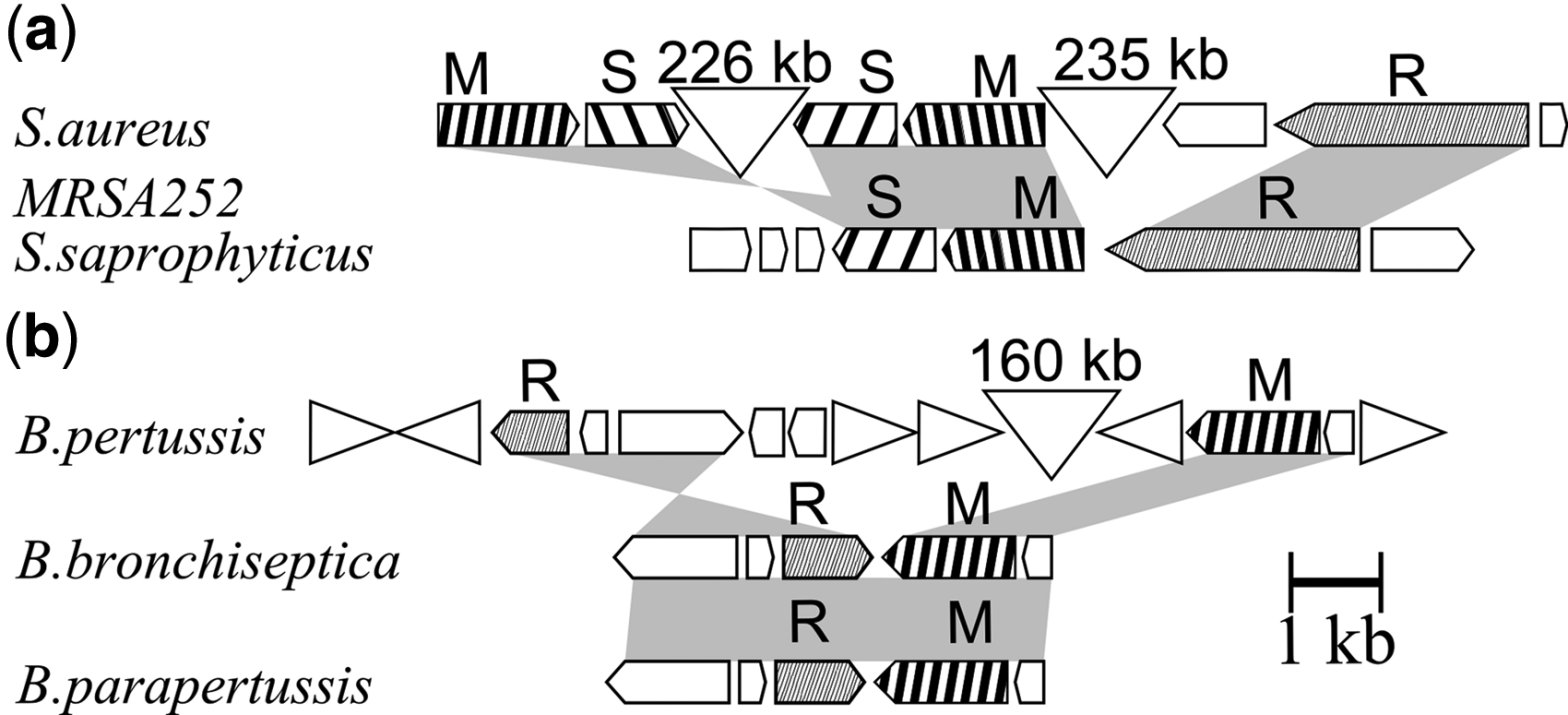
Examples of gene organization of separated R-M systems. (**a**) Gene organization of orthologous type I R-M systems (group 8 in Tables 2, S5) from *S. aureus* and *Staphylococcus saprophyticus.* In the figure are presented separated R-M systems from *S. aureus* MRSA252 genome; the genomic context of orthologous R-M systems is similar and not shown here. (**b**) Gene organization of orthologous Type II R-M systems (group 1 in Tables 2, S5) in the genomes of *B. pertussis, B. bronchiseptica* and *B. parapertussis*. Orthologous genes are connected by gray areas, the genes coding for MTases are shown by arrows with black-and-white strips, the genes coding for REs are shown by light gray arrows, other ORFs are shown by empty arrows, extended inserts between genes are marked by triangles with specified length in kb, and IS-481 elements are shown as triangles with one vertical edge.

Figure 2 shows examples of gene organization in predicted separated R-M systems. Group numbers are the same as in Table 2.

### Separated R-M systems of Type I

Table 2 shows that groups of orthologous R-M systems numbers of orthologous complete R-M systems in the groups of Type I more than group of orthologous R-M systems of Type II.

We identified 38 solitary REs of Type I, as forming separated R-M systems. The functionality of three of them (group 8) has been confirmed experimentally (group 8, see Figure 2a) (9).

In the majority of separated Type I R-M systems, REs genes are separated from linked MTase and S-protein genes. We have some pros and cons on the idea that these genes are actually RE and corresponding separated systems are functional. All groups of orthologous R-M systems of Type I that contain separated R-M systems also include ordinary annotated R-M systems (Table 2) and proteins of separated R-M systems are conservative (see Supplementary Alignments). In the same time, it is known (21) that even high sequence similarity cannot ensure that protein is functional, thus some of the predicted systems can be non-functional. In case of Type I RM systems we can assume that selected proteins form R-M system, but its activity should be experimentally proven, as well as activity of any predicted RM system.

For example, we can look at group 8, which contains several experimentally proven separated R-M systems. It consists of Type I ones from 25 strains of *S. aureus*, one strain of *Anabaena variabilis ATCC 27893*, and one strain of *Oscillatoria sp,* which are included in the orthologous group 8 of 79 R-M systems. Figure 2a shows an example of a separated R-M system of *S. aureus*. The solitary RE gene is located at a distance of more than 200 kb from two cassettes of MTase and S-protein genes, which are also separated by about 200 kb. MTases of these cassettes are very similar (85% of aa identity), but S-proteins contain regions with a low sequence similarity. S-proteins in Type I R-M systems are responsible for specific interaction with DNA. Thus, this case may be interpreted as two hybrid separated R-M systems with different specificities, each composed from one of two cassettes coding for MTase and S-protein and the common solitary RE. This situation can be observed in 23 *S. aureus* genomes.

An R-M system of this type was experimentally studied in three strains of *S. aureus* (*S. aureus* 8325-4, 8325-4, 879R4RF, COL) (9), and was shown to prevent DNA exchange between *S. aureus* and *E. coli* strains.

A separated orthologous RE and only one cassette that included MTase and S-protein were found in four strains: *S. aureus subsp. aureus ST398, S. aureus subsp. aureus JKD 6008*, *A. variabilis ATCC 27893* and *Oscillatoria sp*.

Three *Staphylococcus* species and 26 species of other bacterial and archeael taxons contain ordinary (non-separated) R-M systems that are orthologous to the above-described separated R-M systems (Figure 2a shows an example).

In all 26 strains of *S. aureus*, cassettes of MTase and S-protein genes are localized on the genomic island, similar to those found earlier in several strains (8,22). Solitary RE genes do not possess the repeats, transposones, phage-related proteins or recombination-related genes in the vicinity of 20 kb.

### Separated R-M systems of Type II

We identified putative MTase for 19 predicted solitary REs of Type II. Groups of orhologous Type II systems usually contain fewer members than Type I ones (Table 2), what can be caused by relative diversity of Type II REs. Only two groups of orthologous R-M systems (group 1 and group 2, see Supplementary Table S5) include separated and ordinary complete R-M systems, what can increase the probability that corresponding proteins actually form R-M system. In the same time, as both separated and ordinary R-M systems are actually predicted on sequence similarity basis (14), it is not clear, whether they are functional or not.

Consider, for example putative separated R-M system of Type II from *Bordetella pertussis* (group 1). RE (BpeTORF204P) and MTase (M.BpeTORF740P) genes are separated by about 160 kb (Figure 2b). We identified two orthologous R-M systems in *Bordetella bronchiseptica* and *Bordetella parapertussis*. Both orthologous R-M systems are annotated in REBASE (BbrRORF307P and BpaSORF304P, correspondingly), and they are not separated.

The similarity of aa sequences of REs (>98% of aa identity over 100% of the length) and MTases (>99% of aa identity over 100% of the length) does not leave any doubt on an origin of all three orthologous systems from a very close common ancestor. The functionality of the separated R-M system from *B. pertussis* is supported by a similar gene’s contexts for REs and MTases (Figure 2b), which include a highly conserved upstream region (data not shown).

A big gap between R-M system genes and the change of their orientation in *B. pertussis* can be explained by considerable genomic rearrangements in *B. pertussis* caused by the expansion of insertion sequence elements (family IS481) (23). IS 481 elements are located nearby both MTase and RE genes. The rearrangement is not likely to be genome assembly error. The genome of *B. pertussis* was sequenced with a coverage of 8.9, and RE and MTase genes were located in the center of different contigs (BX640411.1 and BX640413.1).

Supplementary Figure S3 shows other examples of separated R-M system genes organization.

Our predictions about separated R-M systems, having no orthologous ordinary R-M system, are somewhat weaker, because we actually do not know, do the solitary proteins, annotated as REs, have RE activity or not.

All known functional separated R-M systems (9–11) are of Type I. In this work we have found some Type II candidates, but all of them should be experimentally proven.

### Genomic neighborhood analysis of the RE and MTase genes of separated R-M systems

Mobile genetic elements are often (in 38 of 57 cases) located in the 20 kb area to one or both separated R-M system genes (data not shown), similar to what was described earlier for ordinary complete R-M systems (1, 2). This observation supports hypotheses about the origin of separated R-M systems (see Discussion section).

### Solitary REs that do not find candidate cognate MTases

We could not find any candidate cognate MTases for 106 REs (Table 1). From Table 1, we can also see that there are a lot of RE genes with MTase genes located nearby but with the latter annotated in genomes as pseudogenes due to frameshifts or a premature stop codon. For the genes of these REs no putative corresponding MTase genes were found anywhere in their genomes and, thus, they do not participate in separated R-M systems. For all 74 R-M systems that contain a full-length RE and a possibly inactive MTase gene (truncated or with frameshift), we found orthologous R-M systems coded by full-length genes in other organisms (see Supplementary Figure S4 for examples). In the Discussion, we consider possible reasons for the existence of these systems.

From Table 1 we can also see that there are 32 RE genes that have no MTase gene fragments close to them and that do not participate in a separated R-M systems. For these 32 solitary RE genes (and 10 solitary RE genes excluded from the analysis due to the absence of sufficiently close homologs) we identified all MTases of the same Type encoded in the same genome as potential partner MTases (see Supplementary Table S6). We did not found any solitary MTases of the same Type for 11 solitary REs of Type II and 9 solitary REs of Type I. Moreover, we were unable to find any MTases of the same Type for some of them (three REs of Type II and four REs of Type I). Supplementary Table S7 presents these results.

Of these 32 solitary REs, 21 are of Type II. Such REs are of special interest because their presence without MTase should be toxic for a cell. Supplementary Table S8 lists them and shows that all of them are members of orthologous groups with REs from annotated R-M systems. In all cases of R-M systems that contain an RE orthologous to a solitary RE, the MTase gene is located before the RE gene. It is possible that for solitary REs the loss of an MTase gene was accompanied by the loss of some regulatory elements. If true, then the solitary RE genes are not expressed. It is known, however, that in some cases such genes can be transcribed (13); there are no data on whether they are translated or not.

There are nine solitary REs from *Helicobacter pylori* strains (Supplementary Table S8), that belong to four RE orthologous groups. Five of them (members of RE orthologous group 18) are paired to describe earlier Type IV-like REs from cluster 1 (see Supplementary Figure S1). Relatively large number of solitary REs in *H. pylori* genomes would be explained by abundance of R-M systems in these species and frequent genome rearrangements.

Six solitary REs (group 37) from *Bacteroides* are orthologous to five REs from separated R-M systems of orthologous groups 6 and 7. All 11 orthologous REs from *Bacteroides* are quite similar to each other and are annotated as HpaII-like REs. For five of them, we found probable cognate MTases, but for the six remaining we were unable to do so (see Supplementary Table S2). According to REBASE, one of the REs of this group, BthVORF1149P, has no RE activity. It is hard to believe that all these orthologous REs from different species are pseudogenes (13). It is possible that these proteins have changed their function and are not components of an R-M system anymore.

Other possible explanations of solitary RE lacking corresponding MTases are discussed below.

## DISCUSSION

The existence of almost 200 genes coding for proteins with high sequence similarity to known REs that do not have full-sized MTase genes located nearby in the same genome seems to be surprising. All found items are annotated in REBASE as predicted REs. However even if on the basis of sequence similarity it seemed likely that the gene might encode an active RE, this assumption might not be confirmed by experimental verification. Zheng *et al.* show in (21) an example of protein (HindVP from *Haemophilus influenzae*) which is similar (about 40% of aa identity over 90% of the length) to functional RE (for example, HgiDI, BsaHI), but shows no visible restriction activity. Our work based on the speculation that predicted solitary REs can be active, that requires experimental confirmation.

Separated R-M systems, i.e., systems containing an RE and an MTase coded in distantly located genes, may explain the existence of a part of the solitary RE genes found. We predicted 57 such R-M systems. Three of them have been found and confirmed experimentally (9). Examples of R-M systems containing closely located RE and MTase genes and separated (or even located on a plasmid) genes of S-protein are also known (10,11). We did not consider them in this work, but their existence confirms that R-M system genes can be unlinked and functional at the same time. Some of our predictions of separated R-M systems are supported by additional analysis of genomic contexts and protein sequence similarities. They seem to be promising for experimental verification.

One can suppose two scenarios of separated R-M systems formation. First, separated R-M systems can appear after genome rearrangements (often due to invasions of transposons or prophages). Separated R-M systems of *B. pertussis* and *S. aureus* could be formed this way because among orthologous R-M systems there are ordinary (non-separated) ones. Second, new separated R-M systems could arise by the horizontal transfer of an RE gene to a genome carrying an MTase gene with the same or wider recognition specificity as the specificity of the RE (1,24). Orphan MTase (Dam, GATC or CcrM-like, GANTC) also can protect DNA against restriction enzymes with the same or longer recognition sequences (25,26). Probably, this mechanism led to the appearance of separated systems from group 3 (see Supplementary Figure S3).

Presumably, separated R-M systems can appear rather frequently, considering a large number of R-M systems (about 2–4 systems per genome on average over 1040 genomes studied) and the abundance of the genome rearrangements (27). A large number of R-M system genes corrupted by genome rearrangements could be expected (28). Although non-functional genes are apparent candidates for deletion from the genome in evolution (13), we found a hundred of truncated solitary RE genes. Many of them were corrupted, most likely by genome rearrangements because near these genes we found mobile genetic elements (data not shown). However, even separated R-M genes keeping their activity can be beneficial for the host and, thus, be retained in descendents. For example, we found a number of closely related separated R-M systems in strains of one species (*S. aureus*).

Apparently, separated R-M systems lose the ability of simultaneous horizontal transfer because the probability of this event for two genes from different parts of a chromosome is essentially lower than for linked genes. Thus, regarding the R-M system as a selfish element (1), the separated R-M systems can be considered as dead end branches of evolution. It can explain the rareness of this form of R-M systems. Indeed, we identified only 57 separated R-M systems in 1040 analysed genomes, compared with about 3000 annotated complete R-M systems in the same genomes. Although our methods of prediction, as well as the REBASE RE gene prediction procedure, are limited and can miss some solitary REs and separated R-M systems, their actual number is not expected to be much higher.

We found candidates for orthologous separated R-M systems in different taxons (Table 2). The separation of genes of a putative ordinary common ancestor R-M system could occur once or several times independently in different branches of evolution. The latter is in agreement with finding unrelated mobile elements near orthologous R-M genes (data not shown). On the other hand, we speculate that bacteria can benefit from just separated R-M systems, because solitary REs can be used against other bacteria by the introduction of a DNA fragment with a solitary RE gene into the cell of competitors. The solitary RE gene LhopHLHKP, located on the plasmid DNA, is a candidate for such a role.

Note that the genes of all MTases predicted in the work from separated R-M systems are solitary ones, although this was not required by the accepted procedure of their identification. We believe that this could explain the function of a part of solitary MTases, which are more frequent in the genomes than are solitary REs (29).

For 74 genes of solitary REs, we observed MTase pseudogenes in their vicinity and no other candidate for corresponding MTases. We suppose that some pseudogenes of MTases with a single frameshift or a premature stop codon could be erroneous annotations and, thus, represent the expressed genes of MTases, which protect a bacterial cell from the RE. One example is MTase MJ1209 from the *Methanocaldococcus jannaschii* MjaVIP R-M system annotated in RefSeq NC_000909 as a pseudogene with two frameshifts. It was experimentally shown, however, that it is actually functional (24) and that both frameshifts are the errors of sequencing. A similar example in the same genome is the MjaIV R-M system with an annotated frameshift in the MTase gene. In the same work, it was experimentally shown that R.MjaIV is active, and thus a frameshift in the MTase gene is probably an error of sequencing as well. Sequencing errors resulting in frameshifts could be rather frequent cases. Yu *et al.* (30) checked 138 *Brucella abortus S19* genes having a frameshift or a premature stop codon. Resequencing of corresponding DNA fragments showed the absence of gene corruption in 109 of 138 cases; frameshifts and premature stop codons were confirmed in 29 genes.

However, we failed to explain all cases of solitary REs by their occurrence in separated R-M systems or by the presence of MTase genes with erroneously annotated frameshift in the genome. Other possible explanations are as follows. First, there is the corresponding MTase gene in the genome, but it was not found in this work. Corresponding MTase can be significantly different from all known MTases. For example, M.NruI had not shown significant sequence similarity to all previously known MTases’ sequences (31). Other possibility is that RE gene is a member of a separated R-M system, but there is no orthologous R-M system in the studied genomes and thus the MTase gene cannot be found by means of the method used. Examples of similar RE genes from R-M systems with different, non-orthologous MTase genes are described in (19).

Second, the solitary RE gene is not expressed, or is expressed, but the product actually has no endonuclease activity due to mutations in the active site, misannotations, or other reasons. For example, Gingeras and Brooks (32) showed that the deletion of the MTase gene of the *Pseudomonas aeruginosa* functional Type II R-M system PaeR7I surprisingly does not lead to cell killing despite the remaining (thus, solitary) RE (32–34). Bacteriophage invasions also were not restricted, showing inactivation of the RE. On the other hand, transfection of a plasmid carrying the MTase gene restored phage restriction (32). Sequencing has shown an absence of mutations in the RE gene (33), allowing the suggestion that both parts of the R-M system are important for a high level of endonuclease activity.

Third, the host genome is prevented from cleavage or fragmentation. Nucleoid organization, like condensed genomic DNA coated by proteins (35), could prevent cleavage; Vasu *et al.* (36) note this as one of the possible reasons for the existence of REs with promiscuous activity. A bacterial genome can contain no recognition sites of the solitary RE. For example, *Pac*I from *Pseudomonas alcaligenes* is a solitary endonuclease without a corresponding MTase. Stoddard *et al.* (37) explained the existence of the bacterium with this solitary RE by the absence of the RE’s recognition sites in the genome of the host strain. Moreover, bacteria having functional solitary REs can survive due to the high activity of DNA reparation systems (38,39) or the presence of enzymes, specifically hydrolyzing RE (40).

## CONCLUSION

The analysis of genomes containing solitary REs has revealed the candidate separated R-M systems, the RE and MTase genes of which are considerably distant from one another. The experimental evidence of their functionality is a challenge for future work.

## SUPPLEMENTARY DATA

Supplementary Data are available at NAR Online: Supplementary Tables 1–8, Supplementary Figures 1–4 and Supplementary Alignments of protein sequences of orthologous groups.

## FUNDING

Funding for open access charge: Russian Foundation for Basic Research [11-04-91340, in part].

*Conflict of interest statement*. None declared.
